# Stratifying antibiotic use metrics by gestational age and first seven days optimizes antibiotic stewardship in neonatal intensive care units

**DOI:** 10.1038/s41372-023-01826-x

**Published:** 2023-11-18

**Authors:** Sevini Hallaian, Kurlen Payton

**Affiliations:** 1 Department of Pediatrics Division of Neonatology, Cedars-Sinai Medical Center, Los Angeles, CA USA; 2 California Perinatal Quality Care Collaborative, Palo Alto, CA USA

**Keywords:** Paediatrics, Infectious diseases

Antibiotic stewardship has become a high priority in neonatal intensive care units (NICU) given the low incidence of culture-proven infection coupled with a greater understanding of risks of antibiotic exposure [1, 2]. Accurate detection of changes in antibiotic use in response to stewardship interventions is key to determining best practices for a given NICU. Most NICUs use an antibiotic use metric that accounts for all antibiotic use over the NICU course, which may prevent effective detection of improvement and hinder antibiotic stewardship efforts [3]. We hypothesized that stratifying this metric by gestational age and by the first seven days after birth may be more sensitive at detecting improvement than using the unstratified metric.

This is a single-center study at a level IV NICU from March 2019 to January 2023. The active intervention phase occurred from March 2021 to January 2023 during an ongoing statewide multicenter antibiotic stewardship quality improvement collaborative. Plan-Do-Study-Act (PDSA) cycles included creation and implementation of separate early-onset sepsis (EOS) guidelines for neonates ≥35 weeks’ gestational age (GA) and <35 weeks’ GA, guided by the 2018 American Academy of Pediatrics recommendations [1, 2]. The primary outcome was Antibiotic Utilization Rate (AUR), defined as the number of days patients receive at least one dose of parenteral antibiotic divided by total patient days. Both unstratified and stratified monthly AURs were captured automatically and compared using p-charts.

**Fig. 1:**
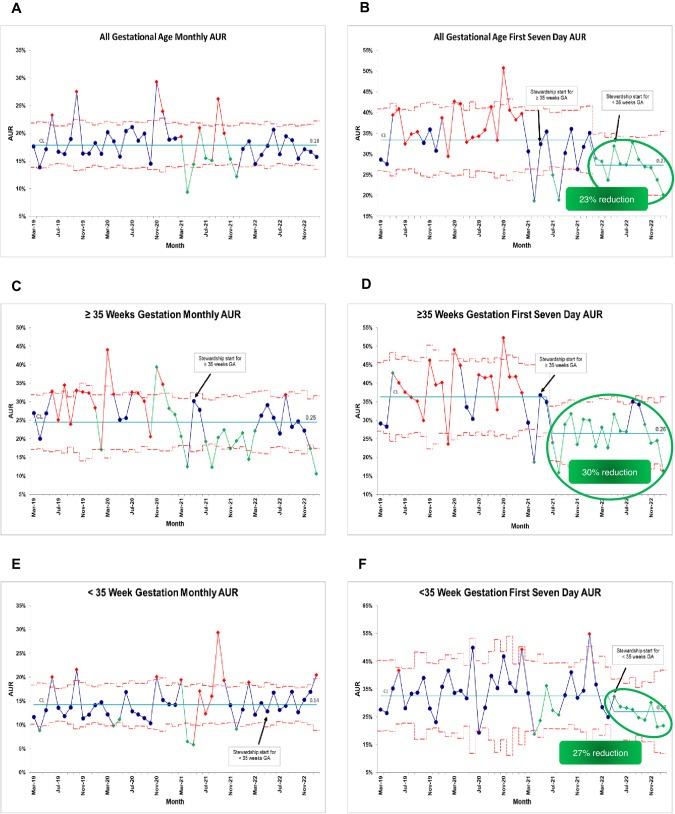
Monthly control charts displaying Antibiotic Utilization Rate (AUR). Unstratified p-chart showing all gestational age (GA) total AUR (**A**) compared to all GA first seven day AUR (**B**); stratified p-chart showing total AUR (**C**) compared to first seven day AUR for ≥35 weeks’ gestation (**D**); stratified p-chart showing total AUR (**E**) compared to first seven day AUR for <35 weeks’ gestation (**F**).

During the study period, a total of 3754 patients were included which accounted for 41,757 patient-days. The first seven day AUR for all babies detected 12 consecutive months of special cause variation (SCV) with a 23% reduction in AUR, while total monthly AUR failed to detect any improvement (Fig. 1A, B). Once stratified by GA, the ≥35 weeks’ monthly AUR detected 9 consecutive months of improvement that was not detected by unstratified AUR. This, however, was not sustained. Further stratification by the first seven days resulted in 13 consecutive months of SCV with a 30% reduction in AUR. This improvement was sustained for 19 months (Fig. 1C, D). The <35 weeks’ first seven day AUR detected 9 consecutive months of SCV with a 27% reduction in AUR, which was undetected by the <35w total AUR nor by total unstratified AUR (Fig. 1E, F).

AUR is a common metric in antibiotic stewardship, yet research on the most optimal approach to its use is limited. Perhaps the difficulty in detecting improvement in AUR lies in a flawed approach to its use. It has been previously stated that the persistently wide range of AURs between NICUs suggests that it is unlikely that one size AUR fits all [4]. In our center, we found that stratifying AUR by GA cohorts and by the first seven days was more sensitive at detecting improvement over time than using unstratified total monthly AUR. We developed separate EOS guidelines for infants ≥35 weeks’ and those <35 weeks’, largely in line with the 2018 American Academy of Pediatrics recommendations [1, 2]. As such, our center approached assessment of their AUR separately. This approach is in line with findings by Flannery, et al, where it was shown that patient-level characteristics such as GA are independently associated with AUR [5]. Similarly, we decided to focus on the first seven days after birth as this is more reflective of EOS stewardship. This also allowed us to capture objective data on provider practices of 36–48 hour ‘rule outs’ versus extended courses to five or seven days. By doing so, we detected significant sustained improvement with 27–30% reduction in the two cohorts, a change that was completely missed using the unstratified metric.

Antibiotic stewardship relies on effectively detecting improvements during rapid PDSA cycles, but unstratified metrics may overlook such improvements and hinder stewardship efforts. Stratifying our metric by GA and/or by the first seven days was more sensitive for detecting improvements in EOS stewardship. NICUs involved in EOS stewardship should consider adopting this method or, alternatively, investigate other variations of metrics which may better support their antibiotic stewardship goals.
